# Supplementary material for: Functionalized Hybrid Iron Oxide–Gold Nanoparticles Targeting Membrane Hsp70 Radiosensitize Triple-Negative Breast Cancer Cells by ROS-Mediated Apoptosis
Source: Cancers (Basel). 2023 Feb 11;15(4):1167. doi: 10.3390/cancers15041167 (PMC9954378; doi:10.3390/cancers15041167)
Supplement: Supplementary file 1 [file cancers-15-01167-s001.zip › cancers-2201221-supplementary.pdf]

Supplementary Materials: Functionalized Hybrid Iron Oxide-Gold Nanoparticles Targeting Membrane Hsp70 Radiosensitize Triple-Negative Breast Cancer Cells by ROS-mediated Apoptosis

Zhiyuan Wu, Stefan Stangl, Alicia Hernandez-Schnelzer, Fei Wang, Morteza Hasanzadeh Kafshgari, Ali Bashiri Dezfouli and Gabriele Multhoff

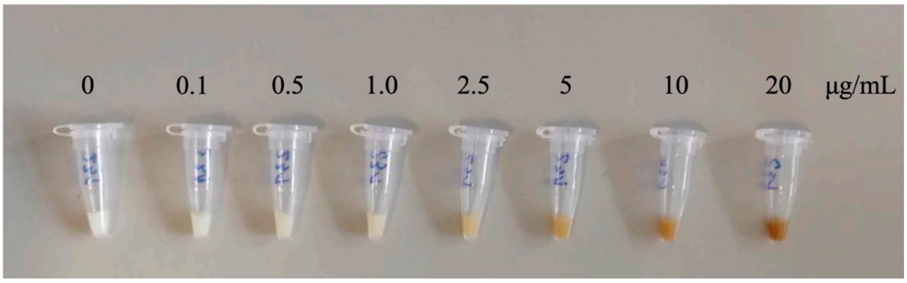

Figure S1. Images of MDA-MB-231 cells cultured with a gradient concentration of TPP-PEG4-FeAuNPs for 24 h. Cell cultured medium were removed and cells were collected in 150 µL tubes.

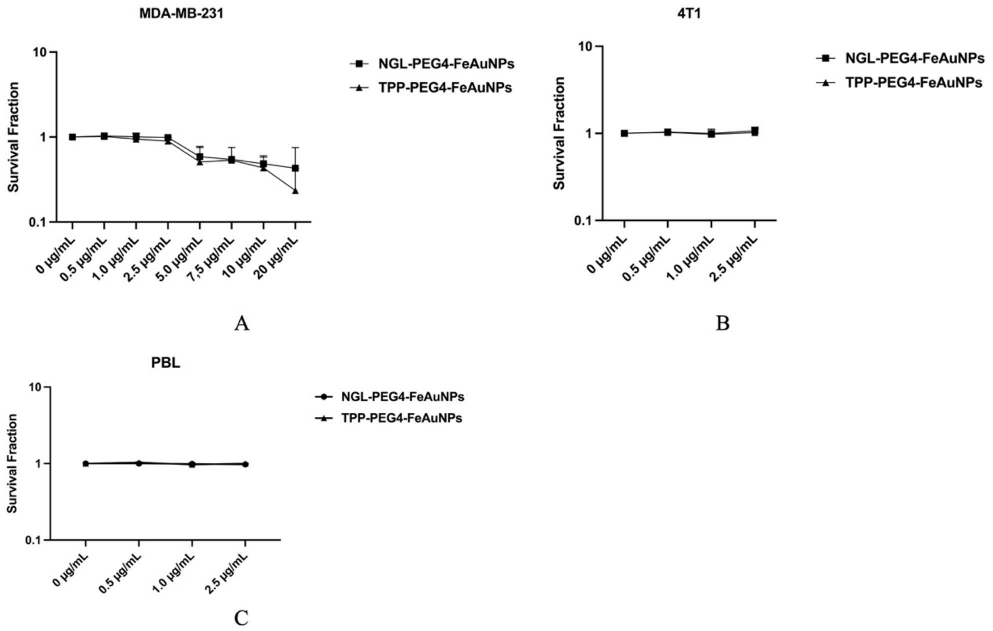

Figure S2. Clonogenic cell survival of MDA-MB-231(**A**) and 4T1(**B**) cells after treatment with NGL-PEG4-FeAuNPs and TPP-PEG4-FeAuNPs in a gradient concentration. CCK-8 assay was used to determine cell viability in peripheral blood lymphocytes (PBL) (**C**) after treatment with NGL-PEG4-FeAuNPs and TPP-PEG4-FeAuNPs in a gradient concentration. Results represent the mean values ± SD of 3 independent experiments.

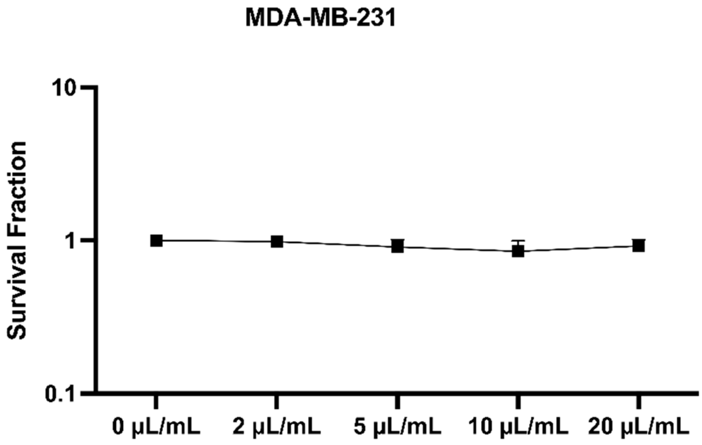

Figure S3. Clonogenic cell survival of MDA-MB-231 cells after treatment with 5mM citrate buffer. Results represent the mean values ± SD of 3 independent experiments.

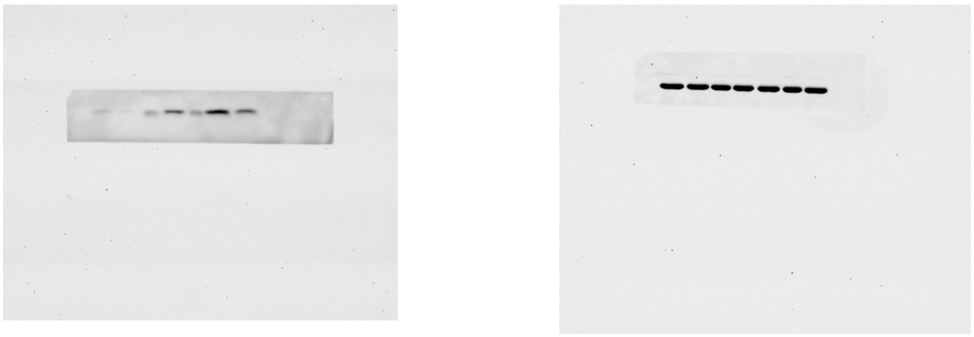

A B

Figure S4. Uncropped Western Blots images for Figue 9. A for γ-H2AX and B for β-actin.
